# Envirotype-based delineation of environmental effects and genotype × environment interactions in Indian soybean (*Glycine max*, L.)

**DOI:** 10.1038/s41598-024-62613-y

**Published:** 2024-05-21

**Authors:** Vennampally Nataraj, Sanjay Gupta, K. H. Singh, Prince Choyal, Raghavendra Nargund, M. Shivakumar, Nisha Agrawal, Giriraj Kumawat, Vangala Rajesh, Rakesh Kumar Verma, Gyanesh K. Satpute, Bairi Srikanth, Savita Kolhe

**Affiliations:** 1 ICAR-Indian Institute of Soybean Research, Indore, Madhya Pradesh 452001 India; 2 ICAR-Indian Agricultural Statistics Research Institute, Pusa, New Delhi 110012 India

**Keywords:** Envirotyping, GGE biplot, Grain yield, Mega-environment and Soybean, Genetics, Plant sciences, Climate sciences, Environmental sciences

## Abstract

Soybean is a rainfed crop grown across a wide range of environments in India. Its grain yield is a complex trait governed by many minor genes and influenced by environmental effects and genotype × environment interactions. In the current investigation, grain yield data of different sets of 41, 30 and 48 soybean genotypes evaluated during 2019, 2020 and 2021, respectively across 19 locations and twenty years’ data on 19 different climatic parameters at these locations was used to study the environmental effects on grain yield, to understand the genotype × environment interactions and to identify the mega-environments. Through analysis of variance (ANOVA), it was found that predominant portion of the variation was explained by environmental effects (E) (53.89, 54.86 and 60.56% during 2019, 2020 and 2021, respectively), followed by genotype × environment interactions (GEI) (31.29, 33.72 and 28.82% during 2019, 2020 and 2021, respectively). Principal Component Analysis (PCA) revealed that grain yield was positively associated with RH (Relative humidity at 2 m height), FRUE (Effect of temperature on radiation use efficiency), WSM (Wind speed at 2 m height) and RTA (Global solar radiation based on latitude and Julian day) and negatively associated with VPD (Deficit of vapour pressure), Trange (Daily temperature range), ETP (Evapotranspiration), SW (Insolation incident on a horizontal surface), n (Actual duration of sunshine) and N (Daylight hours). Identification of mega-environments is critical in enhancing the selection gain, productivity and varietal recommendation. Through envirotyping and genotype main effect plus genotype by environment interaction (GGE) biplot methods, nineteen locations across India were grouped into four mega-environments (MEs). ME1 included five locations viz., Bengaluru, Pune, Dharwad, Kasbe Digraj and Umiam. Eight locations—Anand, Amreli, Lokbharti, Bidar, Parbhani, Ranchi, Bhawanipatna and Raipur were included in ME2. Kota and Morena constitutes ME3, while Palampur, Imphal, Mojhera and Almora were included in ME4. Locations Imphal, Bidar and Raipur were found to be both discriminative and representative; these test locations can be utilized in developing wider adaptable soybean cultivars. Pune and Amreli were found to be high-yielding locations and can be used in large scale breeder seed production.

## Introduction

Soybean is the principal oilseed crop in India with a production of 12.99 million tons in an area of 12.27 million hectares^1^. It contributes around 42.06% to the total oilseed area and 34.45% to the total oilseed production in the country^1^. Nevertheless, its production is impeded with the rainfed mode of cultivation and changing climatic conditions. Development of high-yielding and wider adaptable soybean varieties and environment-specific adapted cultivars is vital to deal with the everlasting edible oil demand in India.

Phenotypic expression of agronomic traits is a result of genotypic and environmental effects (E), and Genotype × Environment interactions (GEI)^2,3^. Soybean grain yield is a complex trait governed by many genes and is confounded with environmental effects and GEI that affect the heritability, leading to the decline in response to selection^4,5^. In Multi-environment trials (METs) of soybean, predominant portion of the total variation is attributed by E and GEI^3,6–8^. Therefore, E and GEI are important factors to be accounted while developing and recommending soybean cultivars across different environments. Envirotyping is a pipeline of collection and processing of raw data on environmental variables and calculation of eco-physiological parameters so as to postulate the development of an organism in a given target environment^9^. Recent advancements in geographic information system (GIS) field and availability of environmental big data are the avenues for better understanding and handling of environmental effects in crop breeding trials for the maximization of selection gain and prediction accuracy^10^. Environmental characterization of a crop growth period can help in defining the driving factors affecting the adaptation and yield manifestation in a particular location^11^. Genotypic plasticity across the environmental gradient during crop cycle results in GEI^12^. A Mega-environment is a group of homogenous locations with same winning genotypes and minimum or non-cross over GEI^13^. Repeatable part of GEI can be dealt through mega-environments through breeding for ME-specific varieties. On the other hand, unrepeatable GEI can be handled through selections and evaluations within a ME^14^. Most common method of identifying MEs is which-won-where pattern in GGE biplot analysis^15^. GGE biplot analysis has been employed in ME identification in different crops^14,16–19^ including soybean^20,21^. However, realizing the importance of environmental effects, recent studies also employed envirotyping method to delineate mega-environments^22^. In India, although soybean coordinated trails are conducted based on agro-climatic zones, a systemic study to identify mega-environments is required to execute multi-location trials based on biological criteria. In this direction, in the current study, we attempted to delineate the environmental effects on grain yield, to understand the genotype × environment interactions and to identify the mega-environments by integrating environmental effects, genotypic effects and GEI through envirotyping and GGE biplot methods.

## Material and methods

### Multi-location evaluation of genotypes

**Figure 1:**
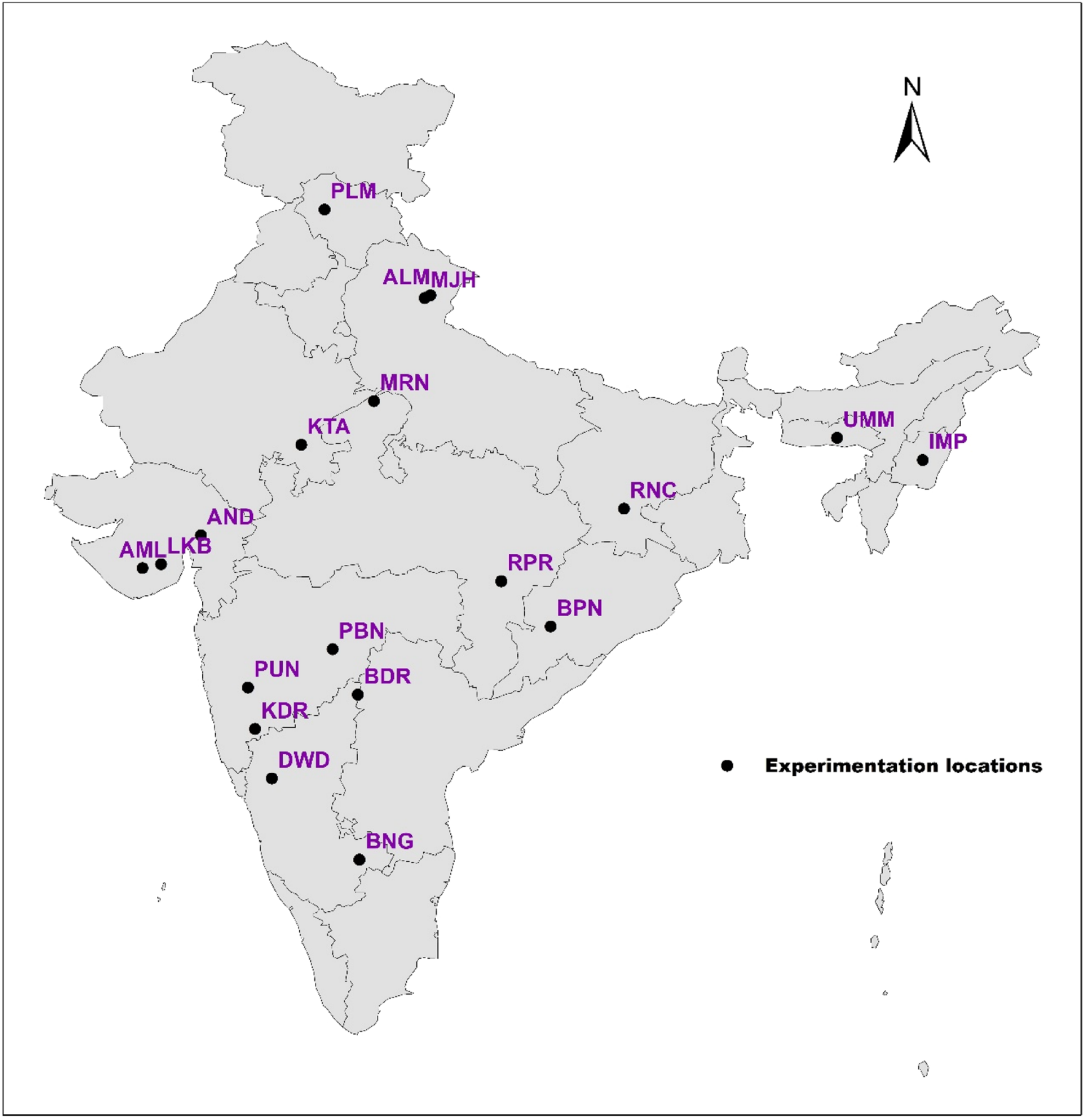
Geographical depiction of test locations under study.

Multi-location evaluation of genotypes was carried out at nineteen locations under All India Coordinated Research Project (AICRP) on soybean (Fig. 1). Genotypes evaluated in each year were different since new entries were being evaluated each year as a part of coordinated varietal development program. A total of forty-five, thirty-two and fifty-two different genotypes were evaluated during 2019, 2020 and 2021, respectively. Details of the test locations are given in Table 1 and details of genotypes evaluated are given in Tables S1–S3. Trials were conducted in Randomized Complete Block Design (RCBD) with three replications each in a plot of size 4.05 m^2^. Standard crop production and protection practices were followed. Grain yield was recorded at R_8_ growth stage^23^, and was converted into Kg/ha.

**Table 1:** Basic information on test location under study.

Code	Location	LON	LAT	alt	Ecosystem
ALM	Almora	79.646	29.589	1642	Sub humid
AML	Amreli	71.22	21.6015	128	Semiarid
AND	Anand	72.928	22.564	39	Semiarid
BDR	Bidar	77.519	17.91	710	Semiarid
BNG	Bengaluru	77.569	13.081	919	Semiarid
BPN	Bhawanipatna	83.164	19.907	248	sub-humid
DWD	Dharwad	75.007	15.458	750	Semiarid
IMP	Imphal	94.052	24.764	821	Humid
KDR	Kasbedigraj	74.514	16.907	554	Semiarid
KTA	Kota	75.864	25.213	271	Semiarid
LKB	Lokbharti	71.764	21.721	67	Semiarid
MJH	Mojhera	79.474	29.507	888	Humid
MRN	Morena	77.994	26.494	177	Semiarid
PBN	Parbhani	76.785	19.24	390	Semiarid
PLM	Palampur	76.545	32.096	1244	Humid
PUN	Pune	74.306	18.119	556	Semiarid
RNC	Ranchi	85.309	23.3441	651	Humid
RPR	Raipur	81.717	21.2284	293	Sub humid
UMM	Umiam	91.545	25.418	1520	Humid

### Statistical analyses

#### Envirotype-based mega-environment delineation

Envirotype-based mega-environments have been identified using R package “EnvRtype”^24^. Environmental grouping was based on similarity among locations with respect to the long-term weather parameters. Nineteen environmental covariables (EC) over 20 years (2022–2021) were retrived from NASA-POWER (https://power.larc.nasa.gov/) through EnvRtype R package. Crop cycle duration (second fortnight of June to second fortnight of October) was considered within each year. Details of the nineteen environmental covariables in Table 2. Nineteen EC were used to create an envirotype-covariable matrix (W) which was further used to calculate the environmental kinships using the function W_matrix() of the EnvRtype package. Four monthly-periods (15 June–15 July, 16 July–15 August, 16 August–15 September and 16 September–15 October) were considered so as to represent the temporal variation during crop growth period. Hence, a total of 1520 (20 years × 19 variables × 4 intervals) variables attributed to the descriptor of each environment. Using the envirotype covariable matrix (19 environmental rows × 1520 climatic variables’ columns), an enviromic kernel (K_E_) was calculated using the following formula

$${\text{K}}_{{\text{E}}} = {\text{WW}}^{/} /\left[ {{\text{trace}}\;\left( {{\text{WW}}^{/} } \right)/{\text{nrow}}\left( {\text{W}} \right)} \right]$$

**Table 2:** Details of the climatic variables used in the current study, and their mean and range across 20 years.

S. No	Code	Climatic variable
1	T2M	Mean temperature at 2 m height (°C day^−1^)
2	Tmax	Maximum temperature at 2 m height (°C day^−1^)
3	Tmin	Minimum temperature at 2 m height (°C d^−1^)
4	PRECTOT	Total rainfall precipitation during the crop cycle (mm)
5	WSM	Wind speed at 2 m height (m s^−1^)
6	RH	Relative humidity at 2 m height (%)
7	TMDEW	dew-point temperature at 2 m above the surface of the earth (°C day^−1^)
8	LW	Downward thermal infrared radioactive flux (MJ m^−2^ day^−1^)
9	SW	insolation incident on a horizontal surface (MJ m^−2^ day^−1^)
10	GDD	Growing degree-days (°C day^−1^)
11	FRUE	Effect of temperature on radiation use efficiency
12	Trange	Daily temperature range (°C day^−1^)
13	VPD	Deficit of vapour pressure (kPa)
14	SPV	Slope of saturation vapour pressure curve (Kpa °C d^−1^)
15	ETP	Evapotranspiration (mm day^-1^)
16	PETP	Deficit by precipitation (mm day^−1^)
17	n	Actual duration of sunshine (h)
18	N	Daylight hours (h)
19	RTA	Global solar radiation based on latitude and Julian day (MJ m^−2^ day^-1^)

Hierarchical clustering based on ‘average method’ has been applied to K_E_ to identify mega-environments. Further, to understand the interrelationship among the variables, between the variables and grain yield and association of variables with mega-environments, Principal component Analysis (PCA) was carried out using R package “factoextra”^25^.

#### GGE biplot-based environmental characterization

GGE biplot method has been applied to identify environmental groupings and mega-environments based on Genotype and Genotype × Environment interactions using R package “EnvRtype”^24^. Year-wise GGE biplot analysis was carried out using the following model^15^.

$$\overline{Y}_{ij} - \mu_{i} - \beta_{j} = \mathop \sum \limits_{n = 1}^{N} \lambda_{{\text{n}}} \alpha_{{{\text{in}}}} \eta_{{{\text{jn}}}}$$

where $\overline{Y}_{{{\text{ij}}}}$ is the grain yield of the *i*th genotype in *j*th environment, μ is the grand mean, β_j_ is the *j*th environmental main effect, *n* is the number of principal components, λ_n_ is singular value of the *n*th principal component and α_in_ and $\upeta$_jn_ are the scores of *i*th genotype and *j*th environment, respectively, for nth principal component and *ε*ij is the residual associated with genotype *i* in environment *j.*

### Ethial statement

Current study was in accordance with the institutional, national, and international guidelines and legislation.

## Results

### Analysis of variance (ANOVA)

Pooled ANOVA revealed significant G × E interaction (GEI) (P < 0.001) for the grain yield (Table 3). Predominant portion of the variation was explained by environmental main effects (53.89, 54.86 and 60.56% during 2019, 2020 and 2021, respectively), followed by GEI (31.29, 33.72 and 28.82% during 2019, 2020 and 2021, respectively) and genotypic effects (6.75, 4.98 and 7.51% during 2019, 2020 and 2021, respectively).

**Table 3:** Pooled ANOVA for grain yield during 2019, 2020 and 2021.

Source of variation	2019		2020		2021	
	DF	F value (%TSS)	DF	F value (%TSS)	DF	F value (%TSS)
Genotype (G)	40	32.49*** (7.92)	29	31.26*** (4.98)	47	51.56*** (7.51)
Environment (E)	18	574.75*** (53.89)	18	549.61*** (54.86)	18	1050.92*** (60.56)
G × E interaction (GEI)	720	8.34*** (31.29)	522	11.64*** (33.72)	846	9.63*** (28.82)
Residual	1520	– (6.85)	1100	– (0.60)	1786	– (5.63)

*DF* degrees of freedom, *TSS* total sum of squares.

***Significant at p < 0.001.

### Envirotype-based ME analysis

**Figure 2:**
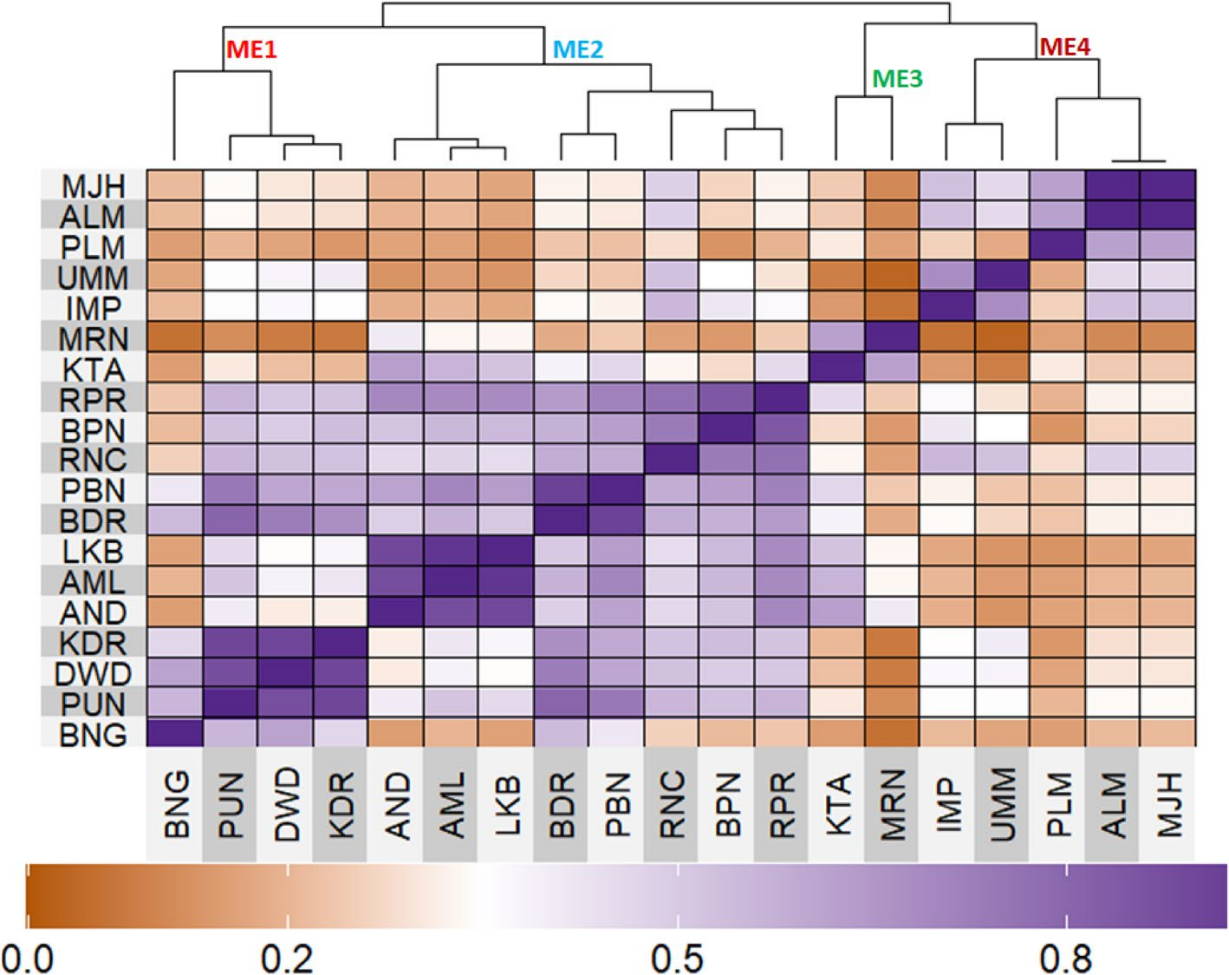
Heat map depicting the delineated mega-environments considering the similarity based on 20 years of information for nineteen environmental covariables.

Through envirotyping, nineteen test locations were grouped into four mega-environments, based on twenty years’ data (2002–2021) on nineteen climatic variables. ME1 included locations—Bengaluru, Pune, Dharwad and Kasbedigraj. ME2 consisted of locations—Anand, Amreli, Lokbharti, Bidar, Parbhani, Ranchi, Bhawanipatna and Raipur. Kota and Morena were included in ME3, while Imphal, Umiam, Palampur, Almora and Mojhera were included in ME4 (Fig. 2).

**Figure 3:**
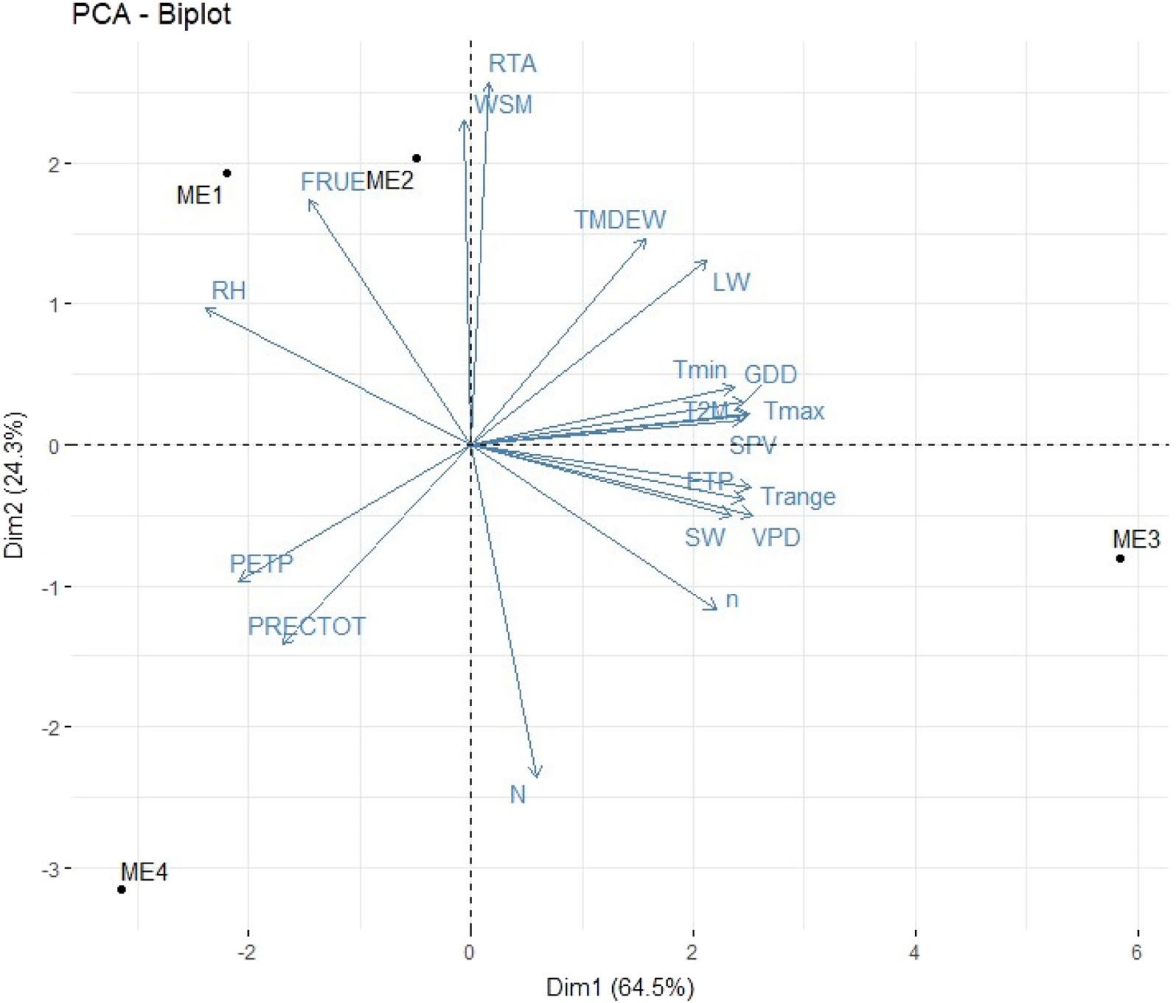
PCA Biplot depicting the inter-relationships among weather parameters based on pooled data of crop cycles over twenty years.

Mean and range of 19 different climatic parameters across twenty years was given in Table S4. Through PCA based on 20-year climatic data, it was found that ME1 was having higher FRUE and RH, while ME2 was mainly characterized by having higher WSM, FRUE and RTA. Climatic variables viz., Trange, ETP, SW, VPD and n were higher in case of ME3, indicating drier environment, and ME4 was associated with higher PETP and PRECTOT, indicating high water availability (Fig. 3).

During 2019, GY was found to be positively associated with RH, FRUE, WSM and RTA, and negatively associated with VPD, Trange, ETP, SW, n and N. During 2020, GY was positively associated with FRUE, RH, RTA and WSM and negatively associated with N, n, SW, ETP, Trange and VPD. Likewise, during 2021, GY was positively associated with FRUE, RH, WSM and RTA and negatively associated with Trange, SW, n and ETP, N, PRECTOT and VPD (Figs. S1–S3).

**Figure 4:**
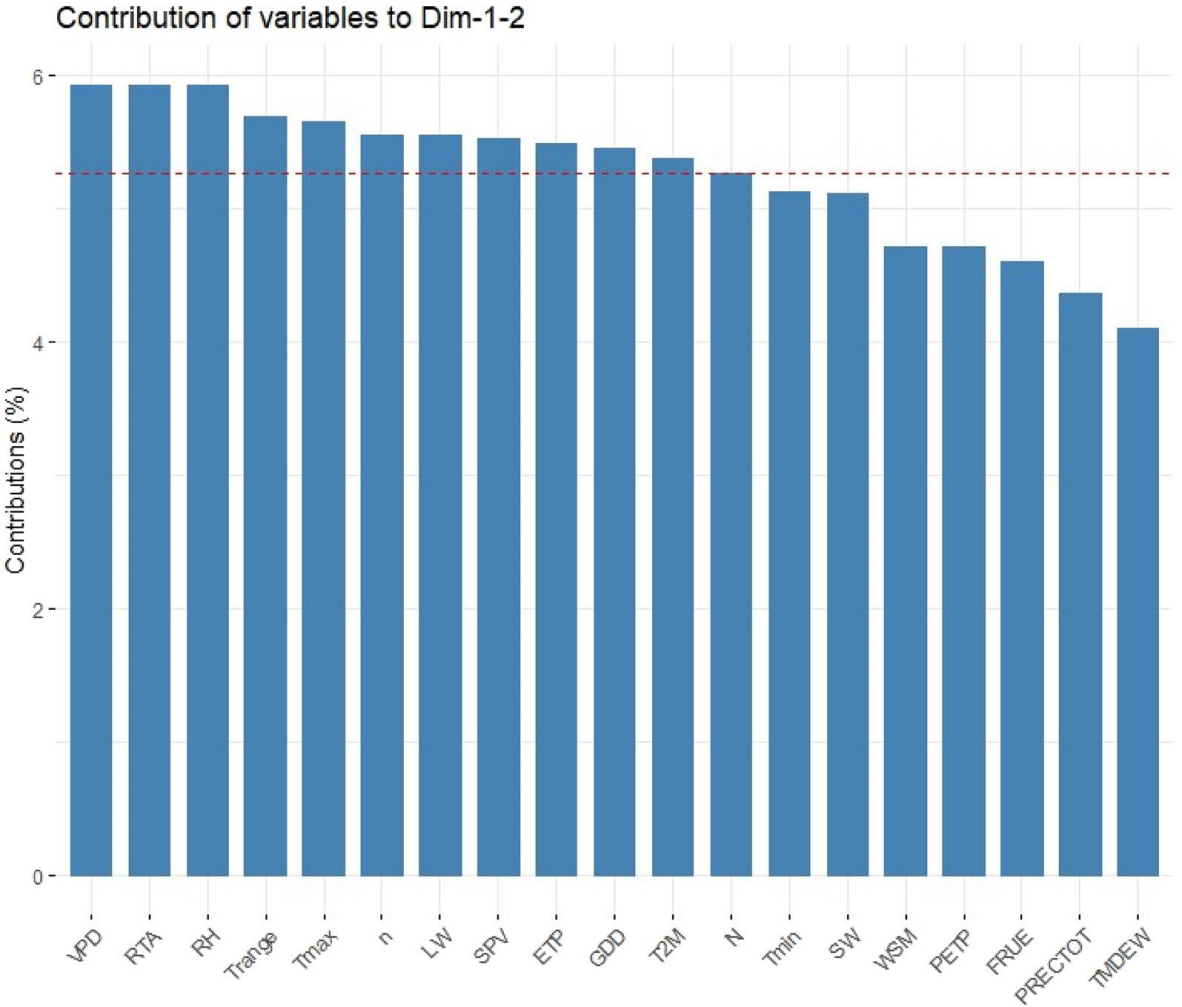
Contribution of individual weather parameters to the variation explained by the first two PCs in 20-year poole data.

Across 20 years, VPD, RTA and RH were the climatic variables that contributed most to the total variation (Fig. 4). During 2019, variation was predominantly attributed by PRECTOT, VPD and LW, while n, RH and VPD contributed most to the total variation during 2020. Likewise, during 2021, Trange, VPD, RTA and RH were the highest contributors to the total variation (Figs. S4–S6).

### GGE biplot-based delineation of mega-environments

**Figure 5:**
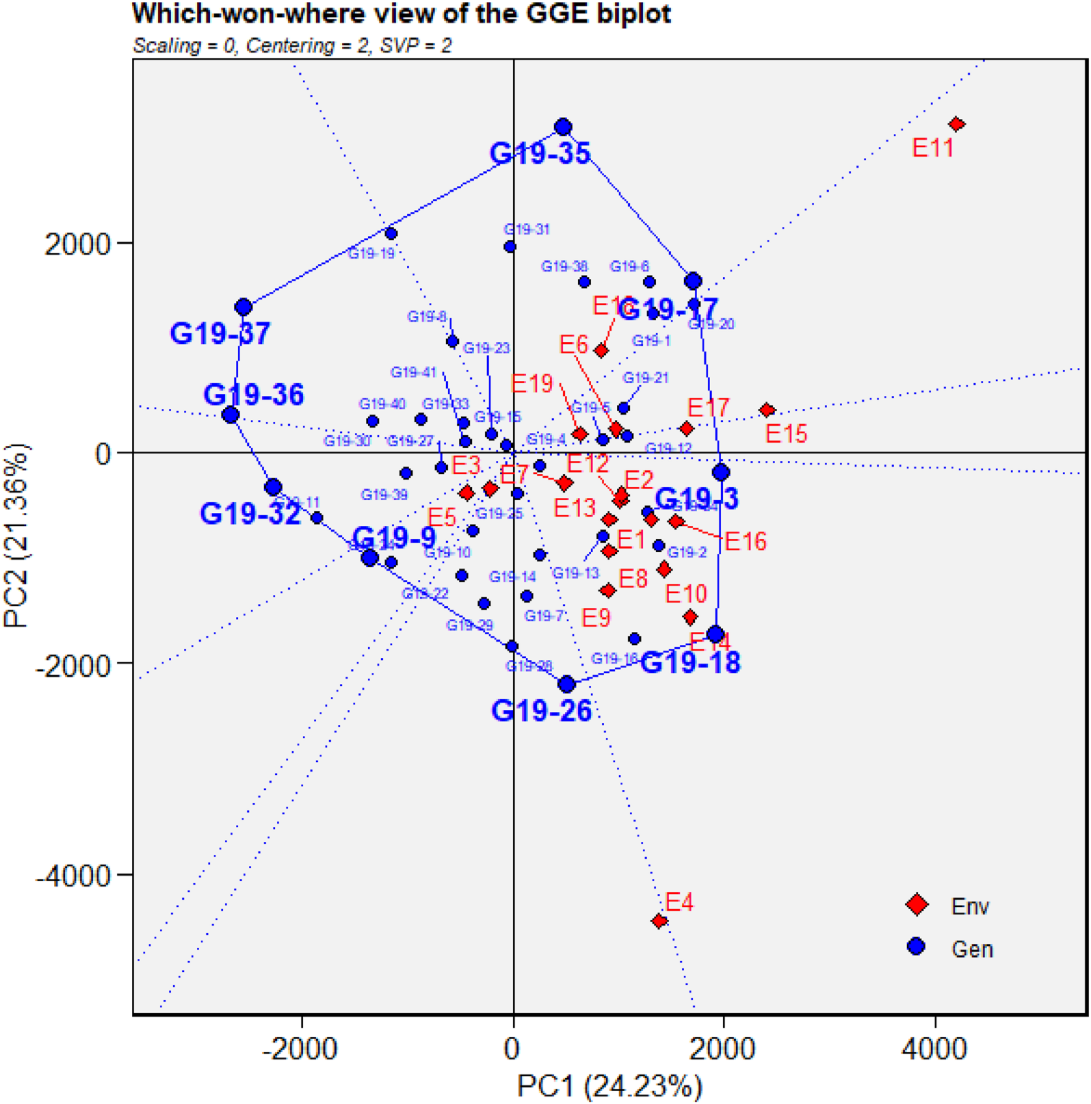
GGE biplot depicting environmental similarity during 2019: E1—Amreli, E2—Anand, E3—Kota, E4—Lokbharti, E5—Morena, E6—Parbhani, E7—Bhawanipatna, E8—Raipur, E9—Ranchi, E10—Imphal, E11—Umiam, E12—Almora, E13—Mojhera, E14—Palampur, E15—Bengaluru, E16—Bidar, E17—Dharwad, E18—Kasbedigraj and E19—Pune.

**Figure 6:**
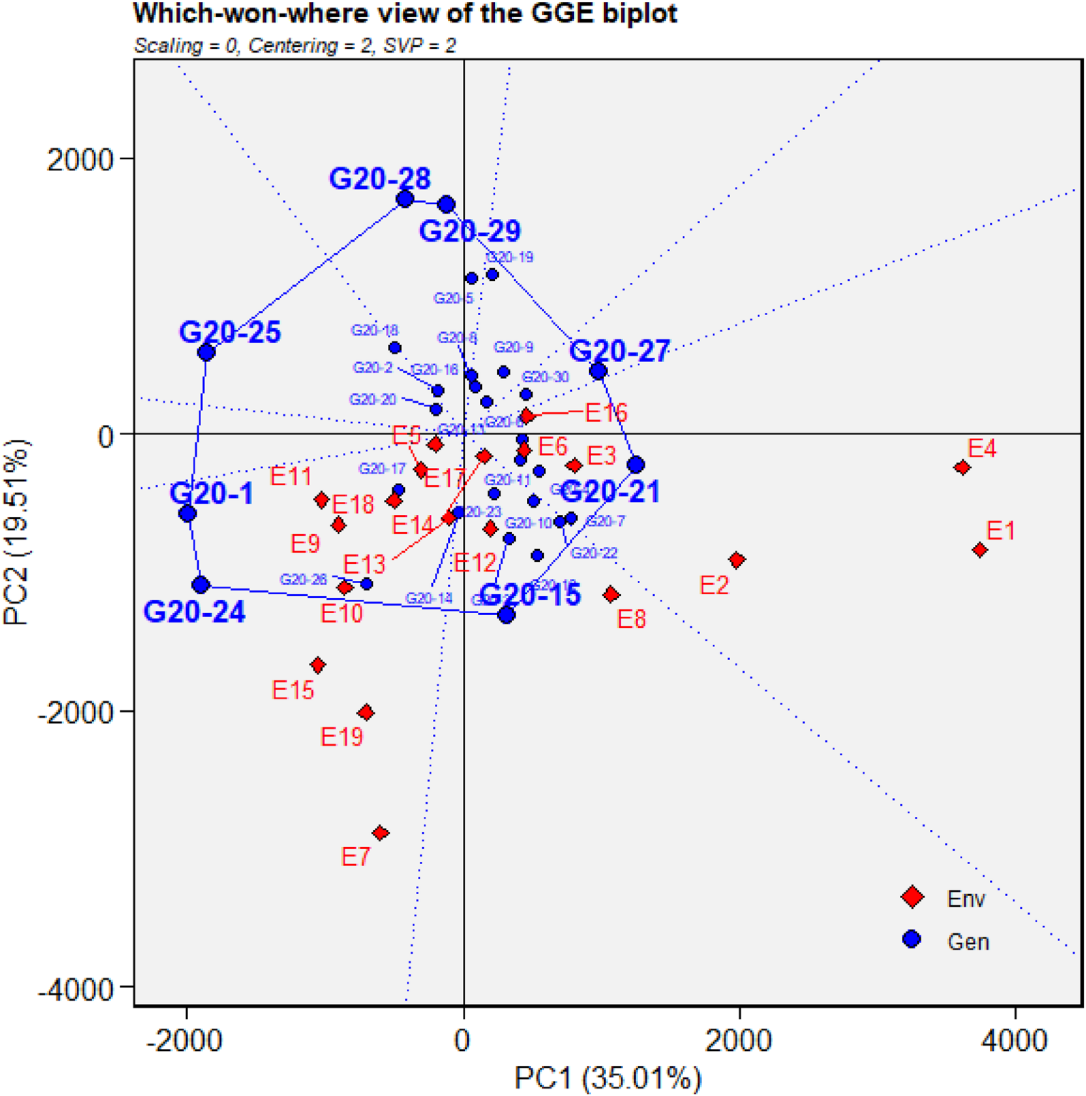
GGE biplot depicting environmental similarity during 2020: E1—Amreli, E2—Anand, E3—Kota, E4—Lokbharti, E5—Morena, E6—Parbhani, E7—Bhawanipatna, E8—Raipur, E9—Ranchi, E10—Imphal, E11—Umiam, E12—Almora, E13—Mojhera, E14—Palampur, E15—Bengaluru, E16—Bidar, E17—Dharwad, E18—Kasbedigraj and E19—Pune.

**Figure 7:**
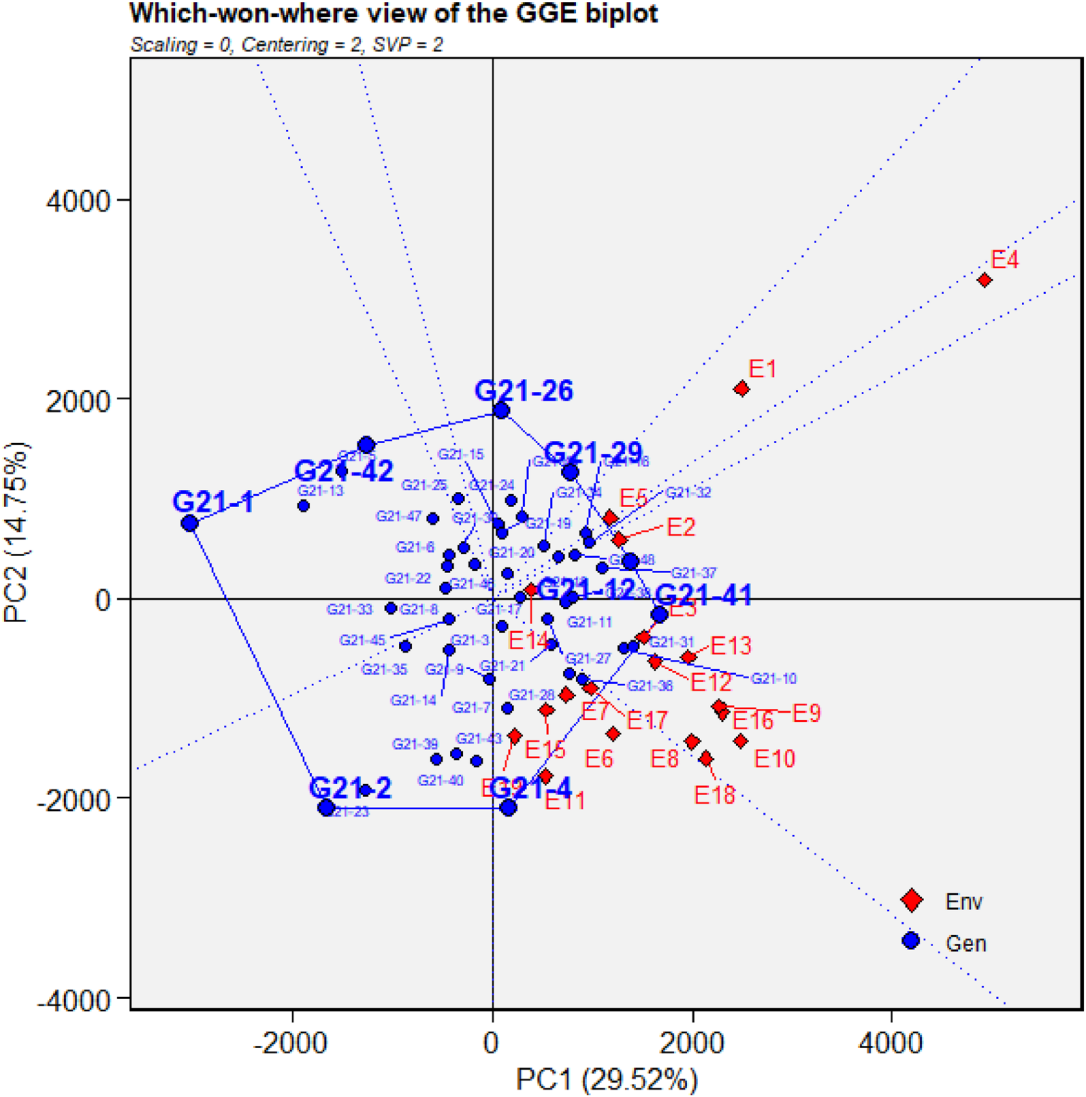
GGE biplot depicting environmental similarity during 2021: E1—Amreli, E2—Anand, E3—Kota, E4—Lokbharti, E5—Morena, E6—Parbhani, E7—Bhawanipatna, E8—Raipur, E9—Ranchi, E10—Imphal, E11—Umiam, E12—Almora, E13—Mojhera, E14—Palampur, E15—Bengaluru, E16—Bidar, E17—Dharwad, E18—Kasbedigraj and E19—Pune.

Grain yield of different sets of genotypes evaluated during 2019, 2020 and 2021 were used in identification of GGE biplot-based MEs. During 2019, nineteen environments were grouped into four MEs. ME1 included Bengaluru, Dharwad, Pune, Umiam and Parbhani. Anand, Amreli, Lokbharti, Bidar, Ranchi, Bhawanipatna, Raipur, Palampur, Imphal, Mojhera and Almora were included in ME2. Kota and Morena constituted ME3, while Kasbe Digraj formed ME4. During 2020, Pune, Bengaluru, Kasbe Digraj, Dharwad, Imphal, Mojhera, Umiam, Ranchi, Bhawanipatna and Morena constituted ME1 while ME2 included Bidar, Parbhani, Lokbharti, Amreli, Anand and Kota. ME4 included Raipur, Almora and Palampur. Likewise, during 2021, Bengaluru, Pune, Umiam, Parbhani, Bhawanipatna and Dharwad were grouped in ME1. Anand, Bidar, Ranchi, Raipur, Almora, Mojhera, Palampur, Imphal, Kasbe Digraj, Dharwad and Kota constituted in ME2. ME3 included Amreli and Morena and Lokbharti formed ME4 (Figs. 5, 6, 7).

### Comparison of ME pattern

**Figure 8:**
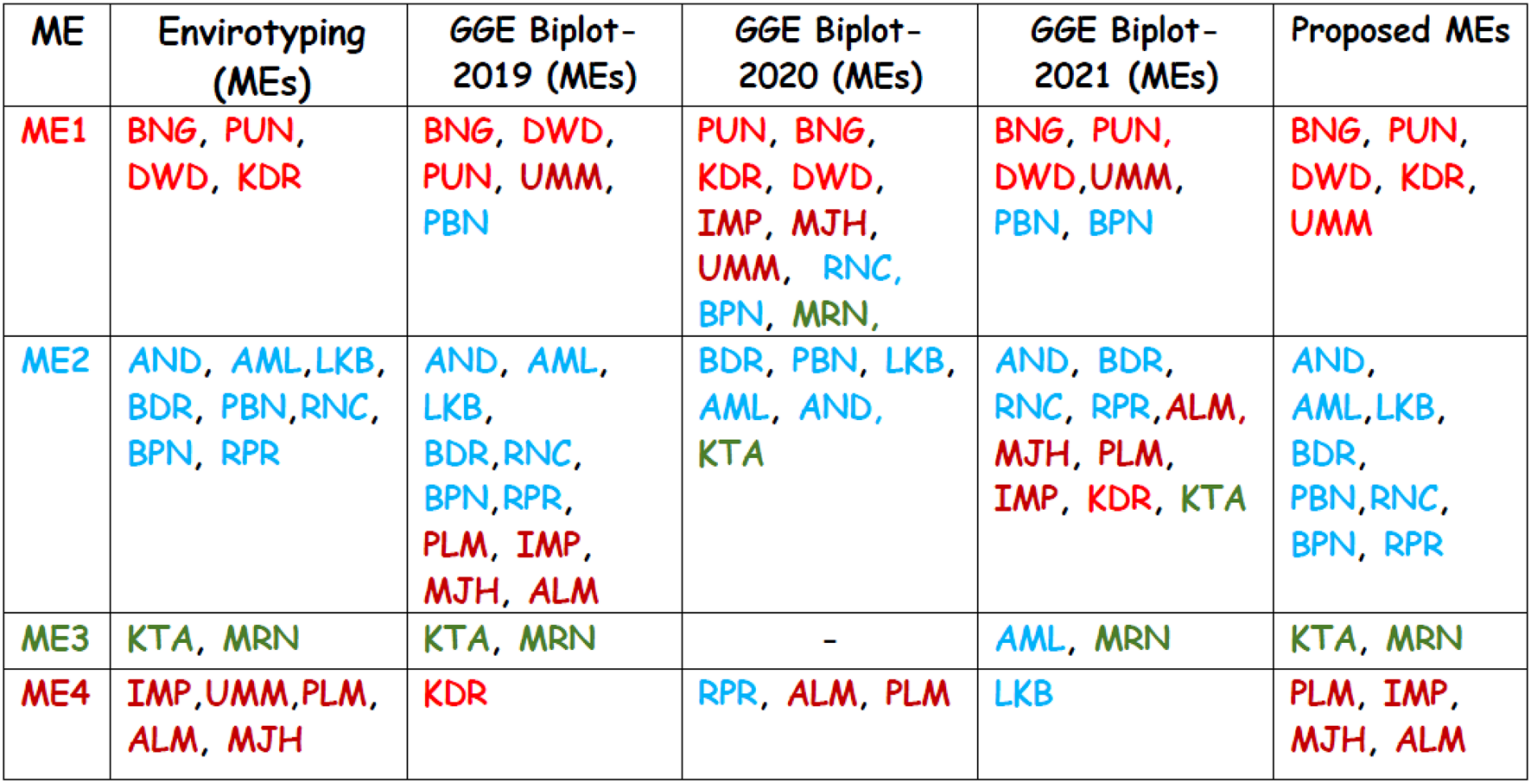
Environmental grouping based on envirotyping and GGE biplot techniques.

Comparison of envirotype based environmental grouping with phenotype based environmental grouping was depicted in Fig. 8. Envirotype based ME1 included locations Bengaluru, Dharwad, Pune and Kasbe digraj (Fig. 2). Among them, through GGE biplot method during 2019, Bengaluru, Dharwad and Pune were grouped together in ME1 while Kasbe digraj was grouped ME4, but the coordinates of these four locations were grouped together, indicating the environmental similarity (Fig. 5). During 2020, all the four locations fell under ME1 (Fig. 6). Similarly, during 2021, though Bengaluru and Dharwad and Pune included in ME1 and Kasbe digraj was included in ME3. However, their coordinates were grouped together, indicating the similarity among them (Fig. 7).

Envirotype based ME2 included Anand, Amreli, Lokbharti, Bidar, Parbhani, Ranchi, Bhwanipatna and Ranchi (Fig. 2). During 2019, all these eight locations were included in ME2 (Fig. 5). During 2020, locations Amreli, Bidar, Anand, Lokbharti and Amreli were encompassed in ME2, whereas, Ranchi and Bhwanipatna were included ME1 and Raipur was included in another ME4. However, Raipur in close proximity with the ME2 in which Amreli, Bidar, Anand, Lokbharti and Amreli were grouped (Fig. 6). Likewise, during 2021, Anand, Bidar, Ranchi and Raipur were grouped together in ME2, while Parbhani and Bhwanipatna were grouped in ME1, Amreali was included in ME3, and Lokbharti was included in ME4. However, coordinates of Parbhani and Bhawanipatna were in close proximity with the ME2 in which Anand, Bidar, Ranchi and Raipur were included (Fig. 7).

Envirotype based ME3 constituted Kota and Morena (Fig. 2). During 2019 these two locations fell within ME3 (Fig. 5). During 2020, Morena and Kota were included in ME1 and ME2, respectively (Fig. 6). Likewise, during 2021, Kota and Morena were included in ME2 and ME3, respectively (Fig. 7). Envirotype based ME4 comprised of locations viz., Imphal, Umiam, Palampur, Almora and Mojhera (Fig. 2). During 2019 and 2021, Imphal, Palampur, Almora and Mojhera were grouped into ME2, whereas, Umiam was fell under ME1 (Figs. 5 and 7). Similarly, during 2020, Imphal, Umiam and Mojhera were positioned in ME1 while the remaining locations were included in ME4 (Fig. 6).

### Proposed mega-environments

Based on the envirotype-based ME analysis and three-year-based GGE biplot analysis, four mega-environments were proposed. ME1 included five locations viz., Bengaluru, Pune, Dharwad, Kasbe Digraj and Umiam. Eight locations—Anand, Amreli, Lokbharti, Bidar, Parbhani, Ranchi, Bhawanipatna and Raipur were included in ME2. Kota and Morena constitutes ME3, while Palampur, Imphal, Mojhera and Almora were included in ME4 (Fig. 8).

### Discriminativesness vs representativeness of test locations

**Figure 9:**
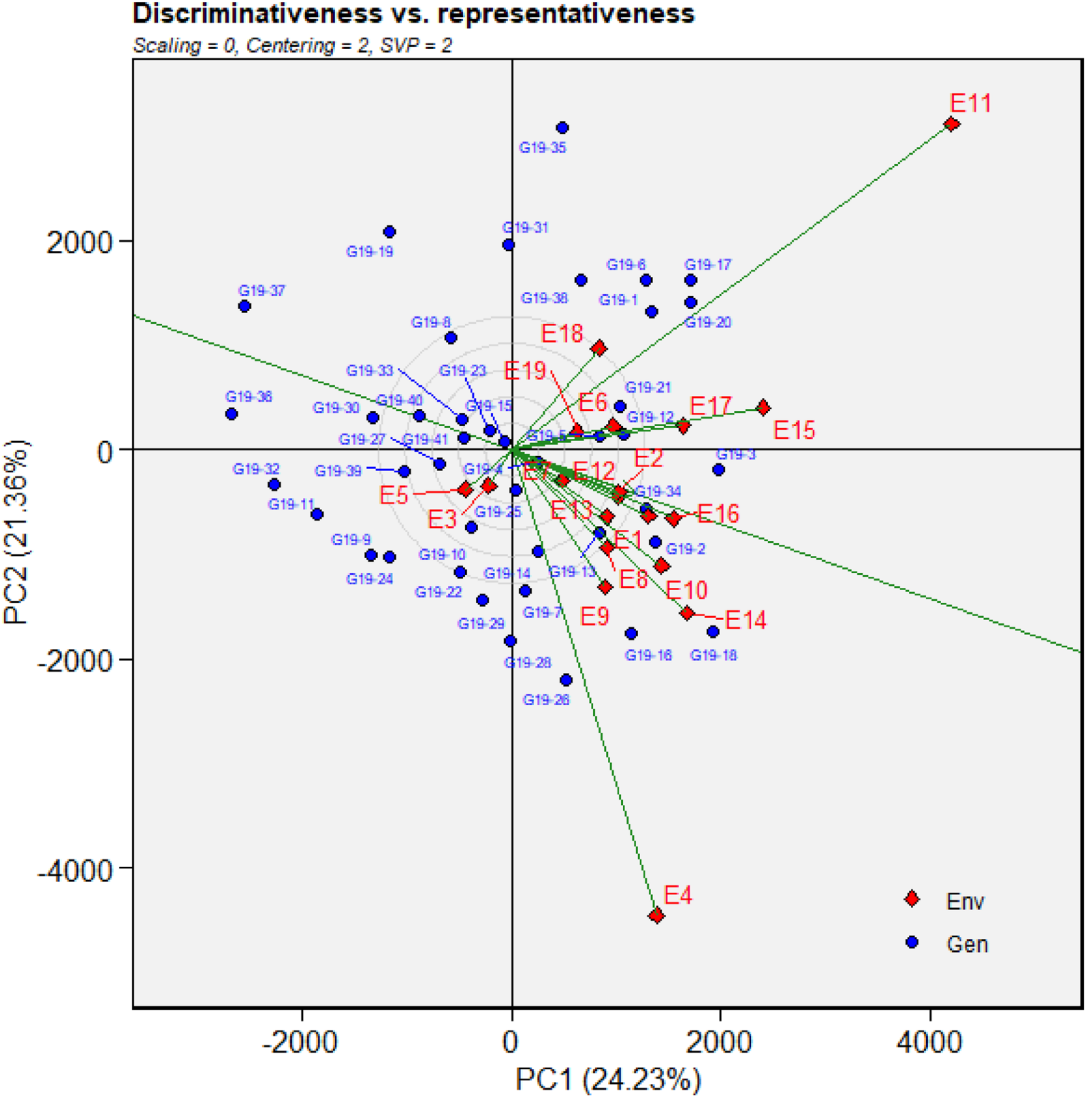
Discrimativeness versus Representativeness analysis of test locations during 2019: E1—Amreli, E2—Anand, E3—Kota, E4—Lokbharti, E5—Morena, E6—Parbhani, E7—Bhawanipatna, E8—Raipur, E9—Ranchi, E10—Imphal, E11—Umiam, E12—Almora, E13—Mojhera, E14—Palampur, E15—Bengaluru, E16—Bidar, E17—Dharwad, E18—Kasbedigraj and E19—Pune.

**Figure 10:**
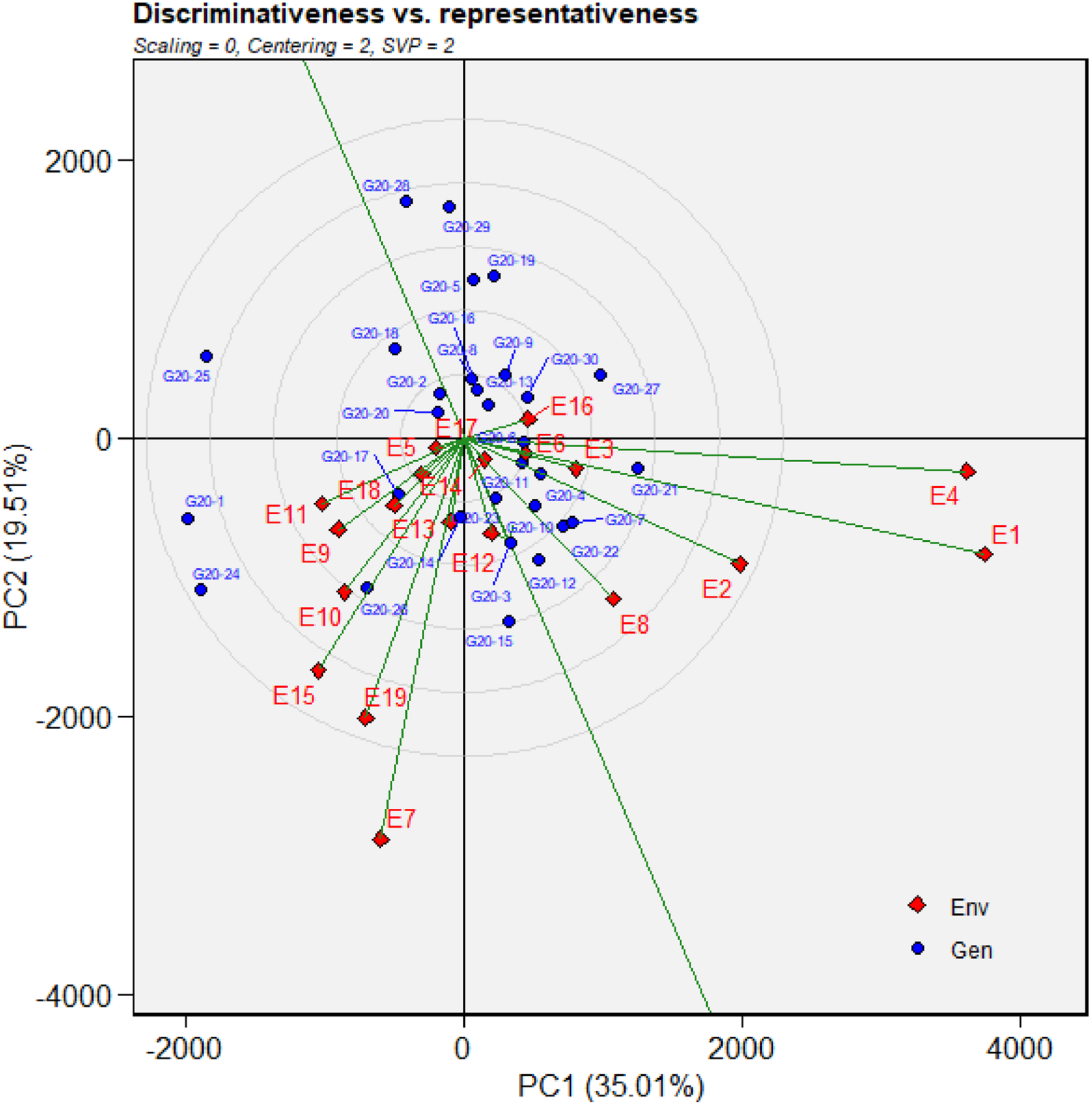
Discrimativeness versus Representativeness analysis of test locations during 2020: E1—Amreli, E2—Anand, E3—Kota, E4—Lokbharti, E5—Morena, E6—Parbhani, E7—Bhawanipatna, E8—Raipur, E9—Ranchi, E10—Imphal, E11—Umiam, E12—Almora, E13—Mojhera, E14—Palampur, E15—Bengaluru, E16—Bidar, E17—Dharwad, E18—Kasbedigraj and E19—Pune.

**Figure 11:**
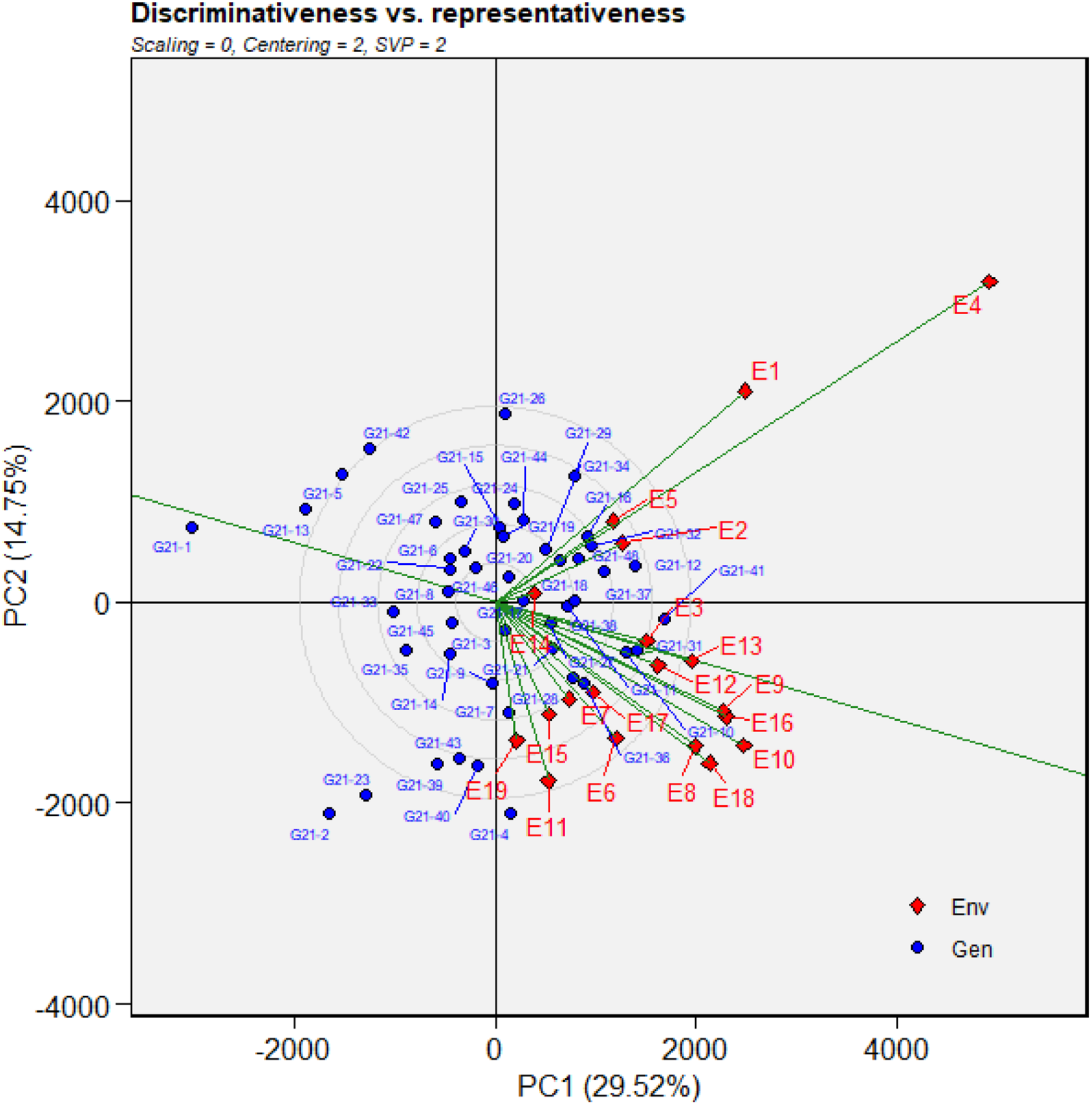
Discrimativeness versus Representativeness analysis of test locations during 2021: E1—Amreli, E2—Anand, E3—Kota, E4—Lokbharti, E5—Morena, E6—Parbhani, E7—Bhawanipatna, E8—Raipur, E9—Ranchi, E10—Imphal, E11—Umiam, E12—Almora, E13—Mojhera, E14—Palampur, E15—Bengaluru, E16—Bidar, E17—Dharwad, E18—Kasbedigraj and E19—Pune.

Based on GGE biplot analysis, during 2019, Lokbharti, Umiam, Palampur and Bengaluru were found to be higher discriminative. Locations Anand, Bidar, Amreli and Alomora were found to be more representative. Bidar, Amreli, Imphal and Palampur were found to be both discriminative and representative (Fig. 9). During 2020, higher discriminating locations were Amreli, Lokbharti, Bhawani Patna and Anand, while representative locations were Almora, Palampur, Mojhera and Raipur. Locations Bhawani Patna, Raipur, Pune and Bengaluru were both discriminative and representative (Fig. 10). Likewise, during 2021, Lokbharti, Amreli, Imphal and Kasbe Digraj were higher discriminative, while representative locations were Mojhera, Kota, Almora and Ranchi. Ranchi, Bidar, Imphal and Raipur were found to be both discriminative and representative (Fig. 11).

## Discussion

Understanding the influence of different climatic factors on yield expression can aid the breeders in better biological interpretation of GEI and to attain genetic progress in a given crop species^11^. Soybean cultivation in India is largely through rain-fed mode, therefore, there is a significant influence of environment and GEI on the genotypic expression of economic and complex traits such as grain yield. Selection gains and productivity ultimately be enhanced through development of high-yielding, wider adaptable and stable cultivars and through development of cultivars specifically adapted to different mega-environments^26^.

Through ANOVA for grain yield, it was found that majority of the variation was contributed by environment effect, followed by GEI interactions, indicating the importance of environment on differential trait expression across environments. Similar findings on predominance of E and GEI were reported by several workers^3,6–8^. Integrating environment with the crop biology will aid in better understanding of genotypic response across locations and in improvising breeder’s decision-making in genotypic choice for adaptation. Nevertheless, not much progress has been made in elucidating the environmental effect on soybean grain yield.

In the current study, enviromics was employed in understanding the effect of different climatic parameters on grain yield. Across three years of field trial, grain yield was positively associated with RH, FRUE, WSM and RTA and negatively associated with VPD, Trange, ETP, SW, n and N.

VPD refers to the vapour pressure difference between the air surrounding the leaves and wet interior of leaves. VPD is the main driving force for transpiration rate (TR) and shows a linear relationship with it^27^. High transpiration rate at increasing VPD decreases the stomatal conductance, which results into the immediate reduction in carbon assimilation as a consequence of decreased CO_2_ influx through stomata^28^. The diminished photosynthesis will penalize the leaf area development and crop growth rate. Leaf area is considered as a major factor in determining crop carbon accumulation and nitrogen storage capacity, ultimately affecting crop yield. High VPD conditions results in downregulation of cell wall development related genes including expansins and extensins, ultimately affecting the leaf expansion and thereby the carbon assimilation^29^.

RH is a very important climatic factor for plant growth as it regulates the photosynthesis and transpiration in plants. It promotes the plant growth and grain yield. High RH conditions limits the VPD and increases the stomatal conductance, and promotes diffusion of CO_2_ inside the stomata while balancing the transpiration rate resulting into high photosynthetic rates and pronounced vegetative growth^30^.

Wind speed (WSM) positively influences the crop productivity. The air flow around the plant canopy causes a significant change in photosynthetic productivity. It minimizes the boundary layer resistance and improves the stomatal conductance and thereby the photosynthesis and carboxylation process^31^. Furthermore, air movement alters the light distribution in the plant canopies, availability of more light to the lower canopies results into significant enhancement in the photosynthetic yield of the plant^32^.

Temperature related parameters under study include T_max_, T_min_, T2M, TMDEW, T_range_, GDD and FRUE. Temperature variables are very crucial in regulating the biochemical and physiological processes in plants. At cellular level, it regulates the enzymatic activities and denatures the enzymes beyond the critical limits. These variables also balance the photosynthesis and respiration processes in plants and thus control the crop productivity. The temperature limits also affects the radiation utilization efficiency of plants (FRUE) and thus affects the photochemical reaction of photosynthesis.

Temperature controls the evapotranspiration process (ETP) and higher air temperature creates the soil moisture stress through increased ETP and thus negatively regulates the grain yield. Further, the daily temperature range (T_range_) which is a potential difference between daily maximum and minimum temperature shows a crop specific response to grain yield^33^. As the higher limits of either of Tmax and/ or Tmin adversely affects the normal physiological and developmental processes. The increased Tmax, results into higher ETP, which creates the moisture, stress and the photosynthetic rates get decreased.

The global solar radiation is the key climatic factor control the grain yield expression across the locations (RTA, in MJ m^2^ d^-1^). Plants absorb the lights of a specific wavelength called photosynthetic active radiation (PAR at 400–700 nm). The availability of sufficient light in PAR region is the primary driving force for crop photosynthesis. Reduction in solar radiation drastically reduces the crop growth and yield through reduced carbon assimilation. It was observed that abundance of solar radiation in western region of China resulted into higher maize grain yield than as compared to eastern region with deficient solar radiation^34^.

The biomass production of any crops depends on light interception and radiation use efficiency^35^. Apart from plant architecture, temperature plays pivotal role in improving radiation use efficiency (RUE). In the current study, effect of temperature on RUE is positively associated with the grain yield. Similarly, in case of maize, higher mean temperature during vegetative period of maize crop improved RUE, thereby the grain yield^35,36^. In case of sorghum, it was found that the low temperatures reduced RUE and yield significantly^35,37,38^.

Though India ranks fifth in soybean production, its average soybean productivity of India is 1200 kg/ha^1^, which is far less than that of major growing countries such as USA, Brazil, China and Argentina. Identification of mega-environments and designing breeding programs aiming at development of cultivars with specific and wider adaption can help in bridging this yield gap. Realizing the importance of environmental effects and GEI on soybean production, based on envirotyping and GGE biplot methodologies, nineteen locations have been grouped into four mega-environments. It was observed that there is no spatial pattern of ME formation, indicating the importance of re-zoning of testing locations in coordinating research programs aiming at development of soybean varieties in India. Locations within a mega-environment may not be necessarily geographically continuous and can sometimes be defined by different forms of biotic and abiotic stresses^13^. Accordingly, in the current study, for example, Umiam location, this is in the north-eastern hill zone, consistently grouped with the southern zone locations. It might be due to the fact that soybean rust (*Phakopsora pachyrhizi*) is predominant in Umiam, Dharwad, Kadbe Digraj and Pune and moderate in Bengaluru^39–42^. Therefore, similarity in this abiotic stress factor may lead to the grouping of Umiam with the southern locations. Additionally, varieties such as soybean MACS 1460 and KDS 753 were identified and released in both southern zone and northern hill zone, indicating the non-cross over genotype × environment interactions across these locations. A detailed investigation on the reasons behind the formation of other geographically non-continuous mega-environments should be done by considering similarity of locations within a mega-environment for different biotic, abiotic, bio-physical, edaphic factors etc. Similar results on non-contiguous formation of mega-environments were reported in sorghum^16^, cowpea^43^ and pearl millet^17^.

In case of GGE biplot analysis, the length of an environmental vector is proportional to its standard deviation from other environments and it determines the discriminating ability of an environment^7^. Among the nineteen environments, Lokbharti and Amreli were found to be consistently discriminative. Unique information on the genotypic performance can be attained from such environments. Environment whose coordinates have smallest angle with Average-Environment Axis (AEA) is considered to be the most representative environment. In the current study, Almora and Mojhera were found to be representative environments, consistently over the years. Environments which are both discriminating and representative are suitable for breeding and selection for wider adaptability. In the current study, Imphal, Bidar and Raipur were found to be both discriminative and representative. Lokbharti was found to be discriminative but non-representative across three years. Such locations can be employed in breeding for specific adaptation and are helpful in identification and culling-out of unstable genotypes^7^. Same information can be obtained from few locations if many of them are closely related. Hence identifying consistently similar environments will help in prioritizing the location choice in multi-location testing (MLT thereby reducing the cost of MLT^16,21^. Consistently high-yielding locations such as Pune and Amreli can be employed in estimating the fullest yielding potential of newly released varieties and seed production to meet the farmer’s seed demand.

## Conclusion

Soybean grain yield is significantly influenced by environmental effects and Genotype × Environment intreactions. In the current study, through envirotyping and GGE biplot methods, nineteen locations across India were grouped into four mega-environments: ME1 included five locations viz., Bengaluru, Pune, Dharwad, Kasbe Digraj and Umiam. Eight locations—Anand, Amreli, Lokbharti, Bidar, Parbhani, Ranchi, Bhawanipatna and Raipur were included in ME2. Kota and Morena constitutes ME3, while Palampur, Imphal, Mojhera and Almora were included in ME4. It was found that many locations which are geographically apart and agro-ecologically different, yielded similar information and were included in the same mega-environment. This indicates the importance of other biological, biophysical and edaphic factors in environment-grouping. To develop specifically adapted varieties, breeding lines must be selected and evaluated at test locations within these mega-environments. Locations Imphal, Bidar and Raipur were found to be both discriminative and representative; these test locations can be utilized in developing wider adaptable soybean cultivars. High-yielding locations such as Pune and Amreli can be used in large scale breeder seed production. Principal Component Analysis (PCA) revealed that grain yield was positively associated with RH (Relative humidity at 2 m height), FRUE (Effect of temperature on radiation use efficiency), WSM (Wind speed at 2 m height) and RTA (Global solar radiation based on latitude and Julian day) and negatively associated with VPD (Deficit of vapour pressure), Trange (Daily temperature range), ETP (Evapotranspiration), SW (Insolation incident on a horizontal surface), n (Actual duration of sunshine) and N (Daylight hours).

### Supplementary Information

Supplementary Information.

## Data Availability

The datasets used and/or analyzed during the current study available from the corresponding author on reasonable request.
