# Supplementary material for: Envirotype-based delineation of environmental effects and genotype × environment interactions in Indian soybean (Glycine max, L.)
Source: Sci Rep. 2024 May 21;14:11629. doi: 10.1038/s41598-024-62613-y (PMC11109282; doi:10.1038/s41598-024-62613-y)
Supplement: Supplementary file 1 — Supplementary Information. [file 41598_2024_62613_MOESM1_ESM.docx]

**Table S1: Details of the genotypes under study during 2019**

|  |  | Pedigree | Breeding Center |
| --- | --- | --- | --- |
| VLS 99 | 19-1 | - |  |
| SL 1213 | 19-2 | SL 958 x SL 955 | Ludhiana |
| PS 1641 | 19-3 | JS 93-52 x PK 472 | Pantnagar |
| NRC 154 | 19-4 | JS 335 × EC 538828 | Indore |
| RVS 2011-32 | 19-5 | JS 335 X PK 1042 | Sehore |
| MACS 1655 | 19-6 | - | Pune |
| SKF 2036 | 19-7 | - | Kashmir |
| AUKS 200 | 19-8 | - |  |
| DS 1318 | 19-9 | P12 X DS 2711 | Delhi |
| NRC 163 | 19-10 | JS 97-52 x EC 538828 | Indore |
| JS 22-01 | 19-11 | SL 738X JS 95-60 | Jabalpur |
| RSC 11-20 | 19-12 | MAUS 504 x MACS 1336 | Ranchi |
| TS 46 | 19-13 | - |  |
| MAUS 717 | 19-14 | Himso 1563 x EC 394837 | Parbhani |
| PS 1642 | 19-15 | JS 97-52 x PS 1225 | Pantnagar |
| AMS 20-19 | 19-16 | Mutant of Bragg | Amravati |
| KDS 1097 | 19-17 | JS 95 60 x MACS 1259 | Kasbedigraj |
| DSb 37 | 19-18 | - | Dharwad |
| ASb 51 | 19-19 | RKS 15 x EC 481309 | Adilabad |
| NRC 149 | 19-20 | NRC94 x SL958 | Indore |
| VLS 98 | 19-21 | - | Almora |
| DS 1326 | 19-22 | P12 X SL 688 | Delhi |
| SL 1234 | 19-23 | SL 783 x SL 871 | Ludhiana |
| RSC 11-22 | 19-24 | JS 97-52 x JS 93-05 | Ranchi |
| Himso 1690 | 19-25 | Hara Soya x Pb 1 | Palampur |
| BAUS 103 | 19-26 | JS-335 x MACS-58 |  |
| JS 22-07 | 19-27 | SL 738 X JS 95-60 | Jabalpur |
| TS 107 | 19-28 | - |  |
| MAUS 712 | 19-29 | MAUS 71 x PK 1029 | Parbhani |
| NRC 168 | 19-30 | JS 97-52 x EC 538828 | Indore |
| MACS 1639 | 19-31 | - | Pune |
| DS 1320 | 19-32 | P9712 X DS 2961 | Delhi |
| KDS 1099 | 19-33 | JS 97 52 x EC 390977 | Kasbedigraj |
| AMS 353 | 19-34 | Bragg x TAMS 41 | Amravati |
| DSb 36 | 19-35 | - | Dharwad |
| AUKS 218 | 19-36 | - |  |
| RVSM 2011-77 | 19-37 | JS 20-30 x JS 93-05 | Sehore |
| SKF 6029 | 19-38 | - | Kashmir |
| NRC 167 | 19-39 | JS 97-52 x Cat 2306 | Indore |
| ASb 50 | 19-40 | NRC 2 x P.Soya | Adilabad |
| RVS 2011-76 | 19-41 | JS 20-29 X JSM 275 | Sehore |

**Table S2: Details of the genotypes under study during 2020**

|  |  | Pedigree | Breeding Center |
| --- | --- | --- | --- |
| DSb 38 | 20-1 | DSb 18 x EC 241780 | Dharwad |
| DS 3105 | 20-2 | Pusa 9712 X JS 335 | Delhi |
| CAUMS 2 | 20-3 | Selection from NRC 2012 M-120-B-2 |  |
| JS 22-11 | 20-4 | JS 97-52 X JS (IS) 90-5-12-1 | Jabalpur |
| DLSb 2 | 20-5 | DSb 21 x SL 958 | Delhi |
| RVSM 2012-11 | 20-6 | - | Sehore |
| RSC 11-39 | 20-7 | - | Ranchi |
| AS 15 | 20-8 | (BR 13 x GJS 3) F2- 2013-5-1-3-2 | Adilabad |
| HIMSO 1691 | 20-9 | - | Palampur |
| JS 22-14 | 20-10 | JS 20-53 X JS 20-34 | Jabalpur |
| DS 3144 | 20-11 | DS 2207 X JS 335 | Delhi |
| DLSb 1 | 20-12 | SL 979 x DSb 21 | Delhi |
| NRC 128 | 20-13 | - | Indore |
| VLS 101 | 20-14 | VLS 75 x VLS 69 | Almora |
| RSC 11-35 | 20-15 | - | Ranchi |
| PS 1661 | 20-16 | JS 97-52 x JS 335 | Pantnagar |
| HIMSO 1692 | 20-17 | - | Palampur |
| PS 1670 | 20-18 | PS 1584 x JS 20-69 | Pantnagar |
| NRC 109 | 20-19 | - | Indore |
| MAUS 806 | 20-20 | - | Parbhani |
| RVS 2011-10 | 20-21 | JS 335 X PS 1042 | Sehore |
| MAUS 768 | 20-22 | - | Parbhani |
| MACS 1701 | 20-23 | RKS 24 X MACS 450 | Pune |
| KDS 1096 | 20-24 | - | Kasbedigraj |
| MACS 1691 | 20-25 | MACS 450 X NRC 67 | Pune |
| KDS 1144 | 20-26 | DS 228 x Type 49 | Kasbedigraj |
| BAUS 96-17 | 20-27 | - |  |
| SL 1212 | 20-28 | - | Ludhiana |
| SL 1250 | 20-29 | - | Ludhiana |
| DS 1312 | 20-30 | - | Delhi |

**Table S3: Details of the genotypes under study during 2021**

|  |  | Pedigree | Breeding center |
| --- | --- | --- | --- |
| 21-1 | SKAU-WSB-101 | - | Kashmir |
| 21-2 | KBS 21-1 | - |  |
| 21-3 | MAUS 818 | - | Parbhani |
| 21-4 | MAUS 791 | - | Parbhani |
| 21-5 | AMS 115 | - | Amravati |
| 21-6 | AMS 19-01 | - | Amravati |
| 21-7 | RVS 13-20 | JS 20-29 x JS 93-05 | Sehore |
| 21-8 | RVS 13-15 | JS 20-88 x JSM 196 | Sehore |
| 21-9 | DLSb 4 | Dsb-21 x SL 958 | Delhi |
| 21-10 | DSb 39 | JS 335 x EC 242104 | Delhi |
| 21-11 | AS 40 | G.Soy 1 x AMRS472 | Adilabad |
| 21-12 | AS 24 | JS 335 x GJ3 | Adilabad |
| 21-13 | JS 23-08 | JS 20-29 x JS 93-05 | Jabalpur |
| 21-14 | JS 23-05 | SL 738 x JS 95-60 | Jabalpur |
| 21-15 | DS 1312 | - | Delhi |
| 21-16 | DS 3163 | DS-2205 x DS 12-13 | Delhi |
| 21-17 | DS 3168 | DS-2210 x DS-2411 | Delhi |
| 21-18 | DS 3124 | DS-2615 x DS-2709 | Delhi |
| 21-19 | SL 1282 | (SL 525 x JS 335) x SL 525 | Ludhiana |
| 21-20 | SL 1230 | SL 905 x {(G. soja x Ankur) x SL (E) 28} | Ludhiana |
| 21-21 | HIMSO-1694 | SL-679 x Harasoya | Palampur |
| 21-22 | HIMSO-1693 | Pb-1 x Himsoya | Palampur |
| 21-23 | VLS 103 | VLS 63 x VLS 2007-24 | Almora |
| 21-24 | PS 1605 | - | Pantnagar |
| 21-25 | PS 1689 | JS 93-52 x PK 472 (F9-1) | Pantnagar |
| 21-26 | PS 1682 | PS 1583 x Bragg (F6-1) | Pantnagar |
| 21-27 | RSC 11-48 | MAUS 504 x MACS 1336 | Ranchi |
| 21-28 | RSC 11-42 | JS 97-52 x JS 93-05 | Ranchi |
| 21-29 | BAUS(M)-3 | - |  |
| 21-30 | BAUS-116 | - |  |
| 21-31 | CAUMS 2 | Selection from NRC 2012 M-120-B-2 |  |
| 21-32 | NRC 195 | - | Indore |
| 21-33 | NRC 203 | EC 538828 x NRC 7 | Indore |
| 21-34 | NRC 193 | JS 97-52 x JS 88-66 | Indore |
| 21-35 | NRC 191 | NRC 7 x EC -538828 | Indore |
| 21-36 | NRC 190 | JS97-52 X JS 335 | Indore |
| 21-37 | NRC 189 | Davis x Kaeri 651-6 | Indore |
| 21-38 | RVSM 16-20 | - | Sehore |
| 21-39 | KDS 1175 | KDS-344 X NRC-102 | Kasbedigraj |
| 21-40 | KDS 1187 | KDS-344 X NRC-101 | Kasbedigraj |
| 21-41 | KDS 1149 | KDS-228 X Type-49 | Kasbedigraj |
| 21-42 | MACS 1672 | Himso-1563 x Macs-450 | Pune |
| 21-43 | ACS 1735 | Himso -1563 x SL-710 | Pune |
| 21-44 | ASb 15 | - | Adilabad |
| 21-45 | TS 21-2 | TS 80 X AMS 33B |  |
| 21-46 | TS 21-1 | SL 742 X TS 37 (Mutant of DSb-12) |  |
| 21-47 | LOKSOY-1 | Type 49 x EC 538836 | Lokbharti |
| 21-48 | KSS 204 | - | - |

**Table S4: Mean and range of climatic parameters under study over 20 years**

| **S.No** | **Climatic variable** | **Mean** | **Range** |
| --- | --- | --- | --- |
| **1** | T2M (Mean temperature at 2m height (°C d^-1^)) | 25.73 | 22.39 – 32.19 |
| **2** | Tmax (Maximum temperature at 2m height (°C d^-1^)) | 29.94 | 25.45 – 37.95 |
| **3** | Tmin (Minimum temperature at 2m height (°C d^-1^)) | 22.37 | 19.16 – 27.03 |
| **4** | PRECTOT (Total rainfall precipitation during the crop cycle (mm)) | 869.19 | 271.06 – 3409.64 |
| **5** | WSM (Wind speed at 2m height (m s^−1^)) | 2.70 | 0.43 – 10.13 |
| **6** | RH (Relative humidity at 2m height (%)) | 79.83 | 50.12 – 92.87 |
| **7** | TMDEW (dew-point temperature at 2 m above the surface of the earth (°C d^-1^)) | 21.46 | 13.59 – 24.94 |
| **8** | LW (Downward thermal infrared radioactive flux (MJ m^−2^ day^−1^)) | 408.95 | 322.06 – 440.39 |
| **9** | SW (insolation incident on a horizontal surface (MJ m^−2^ day^−1^)) | 16.43 | 12.83 – 20.80 |
| **10** | GDD (Growing degree-days (°C day^−1^)) | 18.15 | 14.70 – 24.48 |
| **11** | FRUE (Effect of temperature on radiation use efficiency) | 0.91 | 0.60 – 1.00 |
| **12** | Trange (Daily temperature range (°C d^-1^)) | 7.57 | 4.70 – 12.29 |
| **13** | VPD (Deficit of vapour pressure (kPa)) | 0.92 | 0.29 – 2.87 |
| **14** | SPV (Slope of saturation vapour pressure curve (Kpa °C d^-1^)) | 0.20 | 0.16 – 0.28 |
| **15** | ETP (Evapotranspiration (mm d^-1^)) | 7.55 | 5.83 – 9.52 |
| **16** | PETP (Deficit by precipitation (mm d^-1^)) | 0.27 | 6.69 – 22.47 |
| **17** | N (Actual duration of sunshine (hrs)) | 5.62 | 4.34 – 7.50 |
| **18** | N (Daylight hours (hrs)) | 12.64 | 12.35 – 12.99 |
| **19** | RTA (Global solar radiation based on latitude and Julian day (MJ m^−2^ day^-1^)) | 37.12 | 36.28-37.33 |

**Figure S1: PCA Biplot depicting the inter-relationships among weather parameters and grain yield during 2019**

**
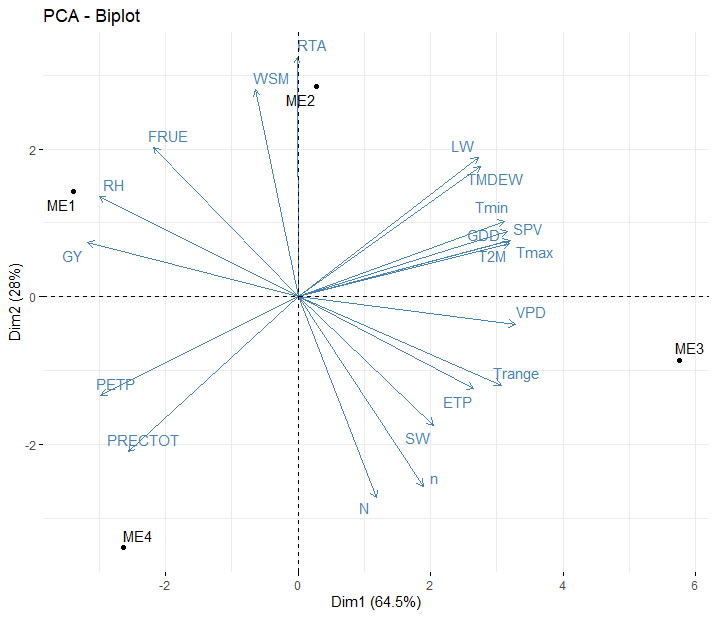
**

**Figure S2: PCA Biplot depicting the inter-relationships among weather parameters and grain yield during 2020**

**
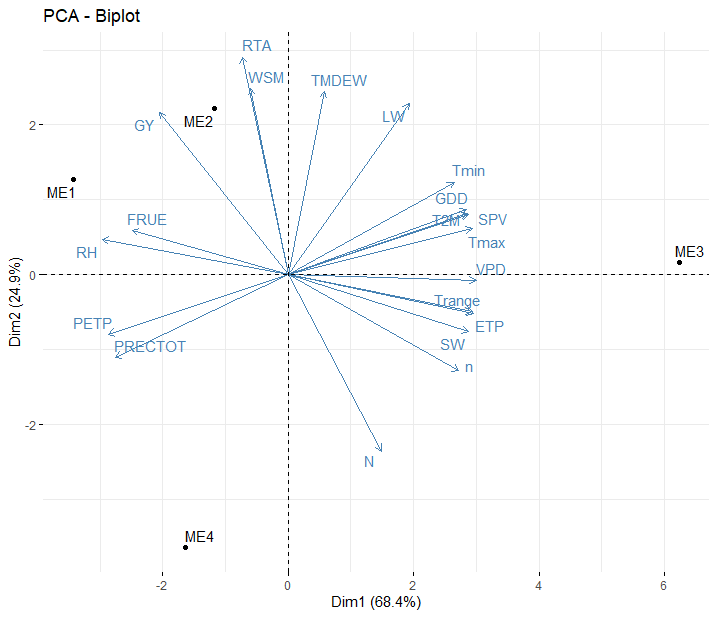
**

**Figure S3: PCA Biplot depicting the inter-relationships among weather parameters and grain yield during 2021**

**
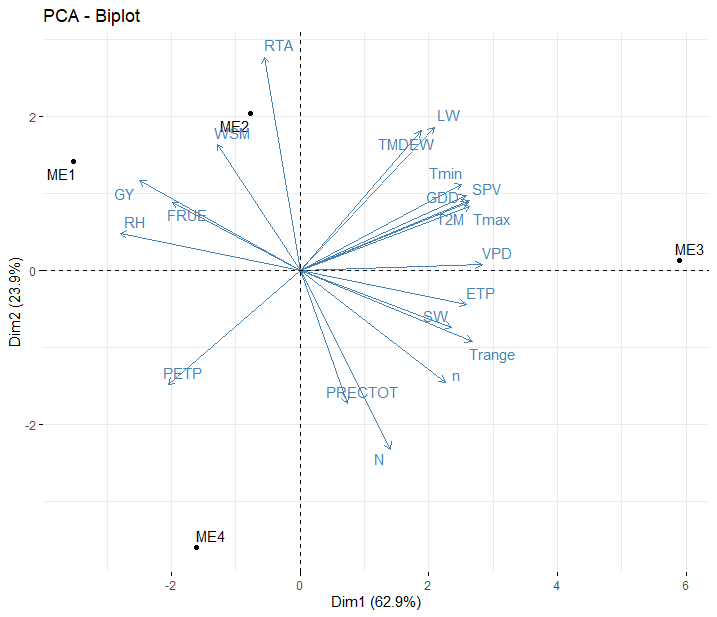
**

**Figure S4: Contribution of individual weather parameters to the variation explained by the first two PCs during 2019**

**
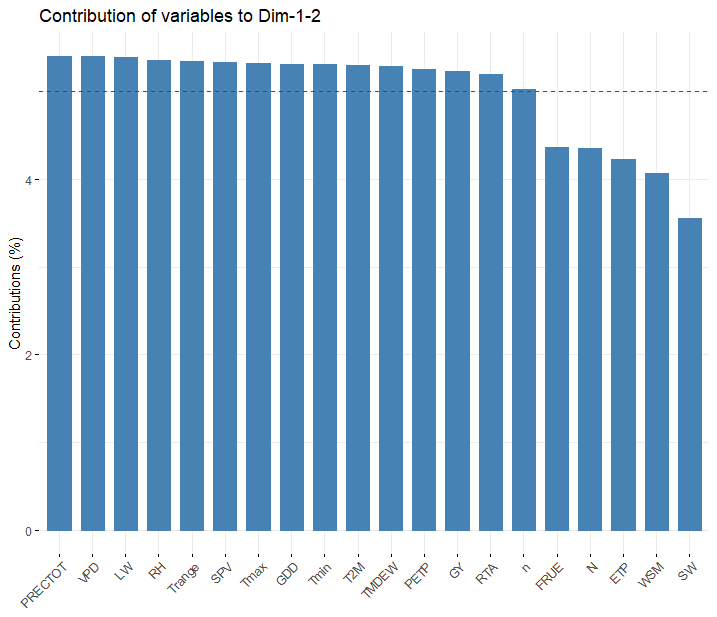
**

**Figure S5: Contribution of individual weather parameters to the variation explained by the first two PCs during 2020**

**
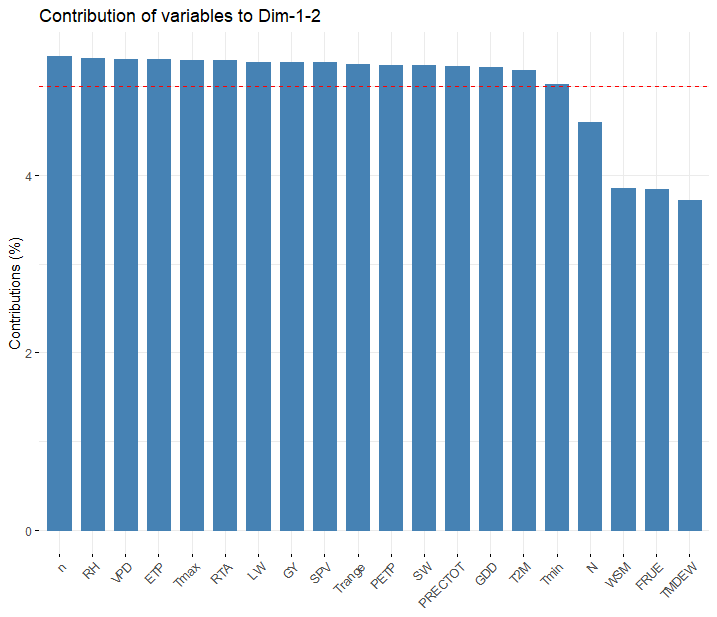
**

**Figure S6: Contribution of individual weather parameters to the variation explained by the first two PCs during 2021**

**
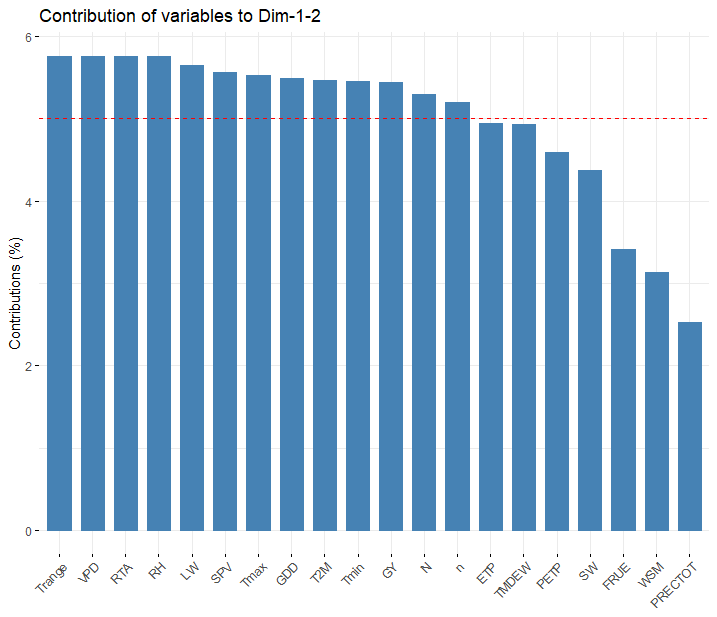
**
